# Oseltamivir-Resistant Pandemic (H1N1) 2009 Virus Infection in England and Scotland, 2009–2010

**DOI:** 10.3201/eid1710.110117

**Published:** 2011-10

**Authors:** Laurence Calatayud, Angie Lackenby, Arlene Reynolds, Jim McMenamin, Nick F. Phin, Maria C. Zambon, Richard Pebody

**Affiliations:** Health Protection Agency, London, UK (L. Calatayud, A. Lackenby, N.F. Phin, M.C. Zambon, R. Pebody);; Health Protection Scotland, Glasgow, Scotland, UK (A. Reynolds, J. McMenamin)

**Keywords:** swine-origin influenza A H1N1 virus, subtype H1N1, virus, antiviral drug resistance, drug resistance, antimicrobial resistance, immunocompromised patient, immunocompromised host, pandemic (H1N1) 2009 virus, H1N1 influenza virus, influenza A virus, research, expedited, England, Scotland, influenza

## Abstract

Oseltamivir has been widely used for pandemic (H1N1) 2009 virus infection, and by April 30, 2010, a total of 285 resistant cases were reported worldwide, including 45 in the United Kingdom. To determine risk factors for emergence of oseltamivir resistance and severe infection, a case–control study was conducted in the United Kingdom. Study participants were hospitalized in England or Scotland during January 4, 2009–April 30, 2010. Controls had confirmed oseltamivir-sensitive pandemic (H1N1) 2009 virus infections, and case-patients had confirmed oseltamivir-resistant infections. Of 28 case-patients with available information, 21 (75%) were immunocompromised; 31 of 33 case-patients (94%) received antiviral drugs before a sample was obtained. After adjusting for confounders, case-patients remained significantly more likely than controls to be immunocompromised and at higher risk for showing development of respiratory complications. Selective drug pressure likely explains the development of oseltamivir resistance, especially among immunocompromised patients. Monitoring of antiviral resistance is strongly recommended in this group.

## Abstract 2

Monitoring of antiviral resistance is strongly recommended for immunocompromised patients.

Neuraminidase inhibitors, antiviral drugs that limit replication of influenza A and B viruses (*1*), are recommended in the United Kingdom for treatment and prophylaxis of patients at higher risk for severe or complicated influenza virus infection (*2*). During the initial containment phase of the 2009 influenza pandemic, antiviral drugs were prescribed for all patients with confirmed infections and their close contacts. During the subsequent treatment phase of the pandemic, the drugs were recommended for persons with suspected influenza virus infections who were at high risk for severe disease (*3*).

Before the 2007–08 influenza season, the development of oseltamivir-resistant influenza was rare (*4*), mainly occurring among persons who were more likely to have prolonged virus shedding, such as children (*5*) and immunocompromised patients (*6*). Patients with subtype H1N1 oseltamivir-resistant strains had the same point mutation in the viral neuraminidase gene (H275Y) that is known to confer high-level resistance to oseltamivir (*7*), but the mutation was associated with reduced infectivity and replicative ability (*8*). During the 2007–08 season, transmissible influenza A (H1N1) viruses resistant to oseltamivir (with the H275Y mutation) emerged and became predominant over susceptible subtype H1N1 viruses (*4*,*9*). The influenza A pandemic (H1N1) 2009 virus was initially reported as fully susceptible to the neuraminidase inhibitors (oseltamivir and zanamivir) but resistant to adamantanes, having the S31N (serine to asparagine) mutation in the M2 ion channel (*10*).

On July 8, 2009, the World Health Organization reported the first sporadic cases of oseltamivir-resistant pandemic (H1N1) 2009 infection in Denmark; Japan; and Hong Kong Special Administrative Region, People’s Republic of China (*11*). By April 28, 2010, a total of 285 oseltamivir-resistant cases had been reported worldwide (*12*), including 45 in the United Kingdom. Three clusters each were reported from Wales (*13*); the United Kingdom; North Carolina, USA (*14*); and Vietnam (*15*). All of the pandemic (H1N1) 2009 oseltamivir-resistant viruses had the previously described H275Y mutation. No reassortment between the pandemic (H1N1) 2009 virus and the seasonal oseltamivir-resistant subtype H1N1 influenza strain has been detected (*16*–*18*), and all of the oseltamivir-resistant viruses have retained sensitivity to zanamivir.

This report describes the epidemiologic, clinical, and demographic characteristics of patients with oseltamivir-resistant pandemic (H1N1) 2009 virus infections in England and Scotland. It also identifies risk factors for severe infection and for the emergence of oseltamivir-resistant virus to inform modifications to current recommendations for the use of antiviral drugs for treatment and prophylaxis of influenza A pandemic (H1N1) 2009 virus infection.

## Methods

### Definition of Case-Patients and Controls

Case-patients were study participants who were hospitalized during January 4, 2009–April 30, 2010, with a confirmed case of pandemic (H1N1) 2009 virus infection with the H275Y mutation in >50% of the virus quasispecies and/or oseltamivir resistance confirmed by phenotyping of virus isolates. Controls were study participants who were hospitalized during January 4, 2009–April 30, 2010, with a confirmed case of pandemic (H1N1) 2009 virus infection with no H275Y mutation detected in the virus.

### Case Detection and Collection of Epidemiologic Information

In the United Kingdom, surveillance of antiviral susceptibility of influenza viruses was performed by the Respiratory Virus Unit (RVU), Health Protection Agency. Pandemic (H1N1) 2009 infection was diagnosed from respiratory specimens by real-time reverse transcription PCR. Regional laboratories refer to RVU specimens from hospitalized case-patients with laboratory-confirmed pandemic (H1N1) 2009. The proportion referred is dependent on several factors. Emphasis is placed on the referral of positive specimens from early and late in the winter season and then a representative number during the peak influenza season. Laboratories are asked to refer equivocal specimens, specimens from patients with clinical antiviral treatment failure, and specimens from immunosuppressed patients and those who died. In addition, a proportion of community respiratory specimens from primary care clinics, selected to provide good regional coverage, were also tested for resistance. Selected specimens were tested by pyrosequencing of the neuraminidase gene to detect the presence of the H275Y mutation (*19*). The results were confirmed whenever possible by culture and phenotyping of virus isolates. Phenotypic antiviral susceptibility was determined by neuraminidase enzyme inhibition assay, using a fluorescent substrate as previously described (*20*). No patients with oseltamivir-resistant pandemic (H1N1) 2009 viruses were identified from Northern Ireland. A hospital cluster in Wales has been described separately (*13*). Therefore, this report only includes cases from England and Scotland.

For all reported cases of oseltamivir-resistant pandemic (H1N1) 2009 virus infection, epidemiologic data were gathered from the responsible clinician by the local Health Protection Unit or by Health Protection Scotland. The following patient information was collected by use of a standardized questionnaire: demographic details, clinical symptoms, complications, outcomes (hospitalization, admission to intensive care unit [ICU], death), underlying medical conditions (chronic respiratory, heart, neurologic, liver, renal diseases, diabetes, immunosuppression, pregnancy), and antiviral treatment.

### Control Group

To identify risk factors for severe disease and for emergence of oseltamivir resistance, a reference control group was defined as hospitalized pandemic (H1N1) 2009 case-patients with virologically confirmed oseltamivir-sensitive infection. The control sampling frame was established by matching all virologically confirmed oseltamivir-sensitive pandemic (H1N1) 2009 specimens diagnosed by the RVU to pandemic (H1N1) 2009 cases reported to a national hospital reporting system.

Through this hospital surveillance system, microbiologists recorded standardized data for all hospital inpatients in England with laboratory-confirmed pandemic (H1N1) 2009 (*21*). Reports were made by 129 of the 160 eligible hospital trusts in England. The dataset included demographic information, underlying medical conditions, antiviral treatment, complications, and information on outcome (ICU admission, death). On the basis of surname, first name, and date of birth, a probabilistic linkage was performed between the 2,817 subtype H1N1 infections recorded in the hospital database and the 3,479 oseltamivir-sensitive pandemic (H1N1) 2009 virus infections confirmed during April 27, 2009–April 30, 2010 (Figure). This method resulted in the selection of 346 study controls. Controls were pandemic (H1N1) 2009 patients infected with oseltamivir-sensitive viruses. All controls had been hospitalized in England and had available clinical information. Recommendations and clinical practice for hospitalization of pandemic (H1N1) 2009 patients were broadly similar in England and Scotland; thus, we assume that this reference group is representative of all pandemic (H1N1) 2009 patients hospitalized in England and Scotland.

**Figure:**
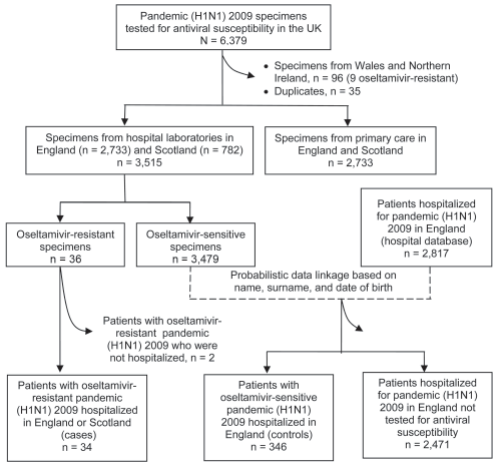
Flow chart showing testing of specimens from persons with confirmed pandemic (H1N1) 2009 infection for antiviral susceptibility, United Kingdom, April 27, 2009–April 30, 2010.

### Study Design and Statistical Analysis

To assess the representativeness of the case-patients whose specimens were tested for antiviral susceptibility and to identify any potential selection bias, our control group was compared with pandemic (H1N1) 2009 patients who were recorded in the hospital database as not having been tested for antiviral susceptibility. To assess differences in distribution of possible risk factors (age, sex, underlying medical conditions) and outcomes, the χ^2^ or Fisher exact test for small numbers was used.

A case–control study was conducted to compare the hospitalized pandemic (H1N1) 2009 patients with oseltamivir-resistant virus infections with hospitalized pandemic (H1N1) 2009 patients with oseltamivir-sensitive virus infections in terms of underlying medical conditions and outcomes. To estimate the association between emergence of resistance and risk factors, we calculated crude odds ratios (ORs) and 95% confidence intervals (CIs). ORs were adjusted for possible confounders by using a step-up logistic regression model. For each variable, missing data were removed from the denominator. Data analysis was performed by using Stata version 11.0 (StataCorp LP, College Station, TX, USA).

### Ethical Approval

This study was conducted under National Health Service (NHS) Act 2006 (section 251), which provides statutory support for disclosure of such data by the NHS and their processing by the Health Protection Agency for the purposes of communicable disease control. Ethical approval was not required, and informed consent was not sought. Health Protection Scotland remains embedded as part of the NHS, in which the sharing of outbreak and investigation data are undertaken as part of their role in the coordination of national outbreaks.

## Results

During April 27, 2009–April 30, 2010, RVU tested 6,379 pandemic (H1N1) 2009 specimens for antiviral susceptibility (*22*). Among 3,515 pandemic (H1N1) 2009 specimens sent by hospital laboratories in England and Scotland, 36 (1%) were oseltamivir resistant and 3,479 (99%) were oseltamivir sensitive (Figure). All samples from primary care clinics were oseltamivir-sensitive.

For the 36 oseltamivir-resistant samples from case-patients, the H275Y mutation was detected by pyrosequencing of the neuraminidase gene. The diagnosis was confirmed by phenotyping for 13 of these patients (36.1%) but was not confirmed by phenotypic typing for the remaining 23 patients due to unsuitable sample type (virus inactivated) or negative culture results. All 36 specimens remained sensitive to zanamivir. Oseltamivir-resistant (H275Y) quasispecies were detected in an additional 13 patients at proportions <50% (the specimen contained a mixture of virus variants, <50% of which harbored the mutation). These patients did not progress to having clinically relevant resistance, and none of the infections could be confirmed phenotypically. For those patients who had further samples available, resistant quasispecies did not persist; thus, these 13 patients are not included further in this study.

Two of the 36 patients with an oseltamivir-resistant strain were not admitted to the hospital: both were immunosuppressed boys who had mild symptoms and recovered. For both patients, the resistant strain developed after antiviral treatment, and a pretreatment specimen (fully susceptible in 1 patient and with <50% of resistant quasispecies in the other) was available.

The remaining analyses relate to the 34 case-patients hospitalized with an oseltamivir-resistant infection who were included in the case-control study. Among these 34 case-patients, 9 (26.5%) were from Scotland and 25 (73.5%) were from England. Symptom onset of case-patients ranged from June 25, 2009, to April 13, 2010, with 3 of the 34 case-patients acquiring their infection during April 27–August 30, 2009, the spring/summer wave of the pandemic.

The 34 case-patients ranged in age from 4 months to 95 years (median 52 years, mean 43.3 years) (Table 1); 23 patients (67.6%) were male, and 11 (32.4%) were female (Table 1). Details of symptoms were available for 22/34 case-patients (64.7%). The most common symptoms were cough (n = 20, 91.0%), fever (n = 17, 77.3%), and dyspnea (n = 12, 54.5%). Rhinorrhea, myalgia, headache, and fatigue were reported for 8 case-patients (36.4%) and gastrointestinal symptoms for 6 (27.3%).

**Table 1:** Distribution and reported associations of age, sex, and underlying medical conditions of study case-patients and controls hospitalized for pandemic (H1N1) 2009, England and Scotland, April 27, 2009–April, 30, 2010*

Patient characteristic	No. (%) case-patients, n = 34			No. (%) controls, n = 346			OR (95% CI)	
	n	With characteristic		n	With characteristic		Crude	Adjusted†
Sex								
M	34	23 (67.6)		346	155 (44.8)		0.4 (0.2–0.9)	0.4 (0.2–1.2)
F	34	11 (32.4)		346	191 (55.2)			
Age group, y								
0–4	34	4 (11.8)		346	64 (18.5)		1	1
5–14	34	4 (11.8)		346	83 (23.9)		0.8 (0.2–3.2)	0.5 (0.1–3.4)
15–24	34	1 (2.9)		346	59 (17.1)		0.3 (0.1–2.5)	1
25–44	34	4 (11.8)		346	74 (21.4)		0.9 (0.2–3.6)	0.5 (0.1–3.6)
45–64	34	16 (47.1)		346	56 (16.2)		4.6 (1.4–14.5)	2.4 (0.5–11.1)
>65	34	5 (14.7)		346	10 (2.9)		8.0 (1.8–34.9)	4.1 (0.5–31.3)
Any underlying condition	30	28 (93.3)		278	164 (60.0)		9.7(2.4–85.5)	
Respiratory	28	7 (25.0)		284	94 (33.1)		0.8 (0.3–2.1)	
Cardiac	28	3 (10.7)		273	12 (4.4)		3.0 (0.5–12.1)	
Renal	28	1 (3.6)		275	11 (4.0)		1.0 (0.0–7.4)	
Liver	28	3 (10.7)		272	3 (1.1)		12.2 (1.5–95.0)	
Neurologic	28	3 (10.7)		269	15 (5.6)		2.3 (0.4–9.1)	
Immunosuppression	28	21 (75.0)		275	19 (6.9)		35.4 (12.7–102.1)	18.1 (6.6–49.9)
Diabetes	28	4 (14.3)		276	6 (2.2)		9.0 (1.7–41.0)	
Pregnancy	28	0		301	19 (6.3)			
Other chronic disease	28	6 (21.4)		258	32 (12.4)		2.5 (0.7–7.2)	

*Case-patients were those with osteltamivir-resistant pandemic (H1N1) 2009 strains; controls were those with osteltamir-sensitive pandemic (H1N1) 2009 strains. n indicates no. patients with information available for that category. OR, odds ratio; CI, confidence interval. †OR adjusted for age, sex, and underlying conditions (e.g., immunosuppression, chronic respiratory diseases).

Of 25 case-patients with information available regarding complications, 21 (84.0%) reported complications: in 19 (76.0%), pneumonia or bronchitis developed, 1 (4.0%) had encephalitis, and 1 (4.0%) had acute renal failure related to secondary group A streptococcal infection. Of the 25 case-patients with available information, 12 (48.0%) were transferred to ICU for 6–31 days (mean 16.9 days, median 15 days).

Thirty case-patients had available information regarding underlying medical conditions, of whom 28 (93.3%) had >1 underlying medical condition: 21 (75.0%) were immunosuppressed, 7 (25.0%) had a chronic respiratory disease, 4 (14.3%) had diabetes, 3 (10.7%) had a chronic cardiac, liver, or neurologic condition, 2 (8.0%) were morbidly obese, and 1 (4.0%) had chronic renal disease (Table 1). All but 2 of the 21 immunosuppressed patients had a hematologic cancer, and 8 of them had undergone hematopoietic cell transplantation (Table 2).

**Table 2:** Type of immunosuppression, presence of hematopoietic cell transplant, and outcomes for patients with oseltamivir-resistant pandemic (H1N1) 2009, England and Scotland, April 2009–April 30, 2010*

Type of immunosuppression	No. with oseltamivir-resistant strains	No. with hematopoietic cell transplant	No. admitted to ICU	No. deaths
Leukemia				
Acute lymphocytic	2	1		
Acute myeloid	3	2	1	1
Chronic lymphocytic	5	2	2	2
No precision	1			
Lymphoma				
Non-Hodgkin	2	1	1	1
Marginal zone	1			
Mantle cell	2			1
Multiple myeloma	1	1		1
Aplastic anemia	1			1
Hematologic cancer with no precision	1	1		
TRAPS	1			
HIV	1		1	
Total	21	8	5	7

*ICU, intensive care unit; TRAPS, tumor necrosis factor receptor–associated periodic syndrome.

Eleven of 30 case-patients (36.7%), ranging in age from 2 to 77 years (median 61 years, mean 51 years), have died; 7 of the 11 patients had a hematologic cancer, and the other 4 had multiple chronic diseases. For 6 patients, death was attributed to pneumonia; 2 had septicemia, and 3 had multiple organ failure.

Information on antiviral treatment was available for 33/34 (97.1%)case-patients. In specimens from 31 of the 33 (93.9%) patients, collected after antiviral treatment, an oseltamivir-resistant strain was detected. A pretreatment, oseltamivir-sensitive specimen was available for 22 of these case-patients. For the remaining 2 case-patients, ages 5–9 years, neither a history of antiviral pretreatment nor contact with a case of influenza-like illness could be found. Both patients were immunocompromised and had influenza-like illness symptoms 2–4 weeks before specimens were collected. Both patients recovered fully.

### Risk Factors for Antiviral Resistance

The 346 controls with oseltamivir-sensitive strains ranged in age from 0 to 103 years (median 19, mean 24); 155 patients (44.8%) were male, and 191 (55.2%) were female (Table 3). Of these controls, 58.9% had >1 underlying medical condition. A chronic respiratory disease was the most common underlying condition (33.1%), and 6.9% of controls were immunosuppressed (Table 3). Of the 364 control patients, 67 (19.4%) had a respiratory complication. Of 205 controls for which information was available, 59 (28.8%) were admitted to ICU; of 322 controls for which information was available, 18 (5.6%) died (Table 3).

**Table 3:** Distribution of age, sex, underlying medical conditions, and outcomes among persons hospitalized for oseltamivir-sensitive pandemic (H1N1) 2009 and persons hospitalized for the disease but not tested for antiviral susceptibility, England and Scotland, April 27, 2009–April 30, 2010*

Characteristic	No. (%) patients with oseltamivir-sensitive strains, n = 346			No. (%) patients not tested for antiviral susceptibility, n = 2,471		χ^2^	p value
	n	With characteristic		n	With characteristic		
Sex							
M	346	155 (44.8)		2,471	1,176 (47.6)	0.95	0.33
F	346	191 (55.2)		2,471	1295 (52.4)		
Age group, y							
0–4	346	64 (18.5)		2,471	479 (19.4)		
5–14	346	83 (24.0)		2,471	463 (18.7)	2.72	0.099
15–24	346	59 (17.1)		2,471	406 (16.4)	0.19	0.663
25–44	346	74 (21.4)		2,471	611 (24.7)	0.29	0.590
45–64	346	56 (16.2)		2,471	391 (15.8)	0.13	0.718
>65	346	10 (2.9)		2,471	121 (4.9)	1.86	0.173
Any predisposing disease	278	164 (59.0)		1,985	1,315 (66.2)	5.67	0.017
Respiratory	284	94 (33.1)		2,198	652 (29.7)	1.41	0.235
Cardiac	273	12 (4.4)		2,170	107 (4.9)	0.15	0.699
Renal	275	11 (4.0)		2,173	69 (3.2)	0.52	0.469
Liver	272	3 (1.1)		2,170	23 (1.1)	0.004	1.000†
Neurologic	269	15 (5.6)		2,181	135 (6.2)	0.16	0.692
Immunosuppression	275	19 (6.9)		1,980	237 (12.0)	6.14	0.013
Diabetes	276	6 (2.2)		2,173	104 (4.8)	3.89	0.048
Pregnancy	301	19 (6.3)		2,216	171 (7.7)	0.75	0.387
Other chronic disease	258	32 (12.4)		1,836	262 (14.3)	0.65	0.419
Complications	346	76 (22.0)		2,471	392 (15.9)	8.15	0.004
Respiratory	346	67(19.4)		2,471	374 (15.1)	4.11	0.043
Cardiac	346	2 (0.6)		2,471	0	14.29	0.015†
Renal	346	8 (2.3)		2,471	33 (1.3)	2.02	0.155
Liver	346	1 (0.3)		2,471	0	7.14	0.123†
Neurologic	346	5 (1.4)		2,471	2 (0.08)	22.78	<0.001†
Otitis	346	0		2,471	1 (0.04)	0.14	1.000†
Other	346	12 (3.5)		2,471	22 (0.9)	16.91	<0.001
ICU admission	205	59 (28.8)		1,542	258 (16.7)	17.69	<0.001
Death	322	18 (5.6)		2,253	78 (3.5)	3.55	0.059

*n indicates no. patients with information available for that category. ICU, intensive care unit. †By Fisher exact test.

Controls with oseltamivir-sensitive strains did not differ significantly by age and sex from the hospitalized pandemic (H1N1) 2009 patients not tested for antiviral susceptibility (Table 3). The proportion of controls with an underlying disease, as well as those who were immunosuppressed, was lower compared with patients not tested for resistance (Table 3). Other underlying diseases were distributed equally between these 2 groups. Our reference group of patients with oseltamivir-sensitive infections, although not randomly selected, thus appears to be representative of patients hospitalized with pandemic (H1N1) 2009 virus infection and, thus, reliable for assessing risk factors associated with the development of an oseltamivir-resistant virus among persons hospitalized with pandemic (H1N1) 2009. However, patients with any complication and those admitted to ICU were significantly more likely to be in the group tested for antiviral susceptibility (Table 3), meaning that this study only allowed an evaluation of the course of disease among patients with the most severe pandemic (H1N1) 2009 virus infection.

Comparison between the case-patients with oseltamivir-resistant virus infections and controls with oseltamivir-sensitive infections showed, on crude analysis, that resistance was more common among middle-aged and elderly men (Table 1). Case-patients were 9× more likely than controls to have an underlying medical condition (95% CI 2.4–85.5), particularly immunosuppression (crude OR 35.4, 95% CI 12.7–102.1). Chronic liver disease and diabetes were also significantly more likely among case-patients (crude OR 12.2, 95% CI 1.5–95.0) than controls (crude OR 9.0, 95% CI 1.7–41.0).

After adjusting for age and sex, which were confounders for underlying disease in the stratified analysis, immunosuppression remained the only variable associated with development of oseltamivir resistance (adjusted OR 18.1, 95% CI 6.6–49.9). The proportions of patients with oseltamivir-resistant strains (31/33, 94.0%) and controls with oseltamivir-sensitive strains (152/170, 89.4%) who received antiviral drugs before a specimen was obtained were not significantly different (adjusted OR 1.7, 95% CI 0.4–6.6).

### Risk Factors for Severe Disease

Case-patients with oseltamivir-resistant strains were at higher risk than controls with oseltamivir-sensitive strains for complications (crude OR 18.6, 95% CI 6.0–76.2), particularly for pneumonia and bronchitis (crude OR 15.8, 95% CI 5.4–55.6) (Table 4). A higher proportion of case-patients than controls were admitted to ICU (52.0% vs. 28.8%), although the difference was not significant.

**Table 4:** Distribution and reported associations of outcomes (complications, ICU admission, death) for study patients and controls hospitalized for pandemic (H1N1) 2009, England and Scotland, April 27, 2009–April, 30, 2010*

Outcome	Case-patients with oseltamivir-resistant strains, n = 34			Controls with oseltamivir-sensitive strains, n = 346			OR (95% CI)	
	n	No. (%) with outcome		n	No. (%) with outcome		Crude	Adjusted†
Any complications	25	21 (84.0)		346	76 (22.0)		18.6 (6.0–76.2)	9.0 (2.4–34.3)
Respiratory complications	24	19 (79.2)		346	67 (19.4)		15.8 (5.4–55.6)	6.6 (1.8–23.3)
ICU admission	23	12 (52.01)		205	59 (28.8)		2.3 (1.0–7.1)	2.3 (0.7–7.9)
Death	30	11 (36.7)		322	18 (5.6)		9.8 (3.6–25.4)	2.2 (0.5–9.5)

*n indicates no. patients with information available for that category. ICU, intensive care unit, OR, odds ratio; CI, confidence interval. †OR adjusted for age, sex, and underlying conditions (e.g., immunosuppression, chronic respiratory diseases).

The proportion of patients who died was 9.8× higher (95% CI 3.6–25.4) among case-patients with oseltamivir-resistant strains than controls (Table 4). However, after adjusting for age, sex, immunosuppression, and chronic respiratory diseases, we found a significantly higher risk for complications, particularly for respiratory complications (OR 6.6, 95% CI 1.8–23.3), remained associated with the presence of an oseltamivir-resistant strain (Table 4).

## Discussion

This report summarizes the clinical and epidemiologic characteristics of one of the largest collections of oseltamivir-resistant pandemic (H1N1) 2009 cases described in the literature. Most of the 34 case-patients hospitalized in England and Scotland during April 27, 2009–April 30, 2010, with oseltamivir-resistant pandemic (H1N1) 2009 were immunocompromised middle aged or elderly men. Selective drug pressure in a particular patient subgroup seems to have been responsible for development of the resistant strain for most case-patients. Furthermore, persons with oseltamivir-resistant pandemic (H1N1) 2009 infection were more likely than those with oseltamivir-sensitive virus infections to develop complications.

This study has several limitations. First, our reference group was a convenience sample of patients hospitalized with oseltamivir-sensitive pandemic (H1N1) 2009. Underlying medical conditions and severe outcomes are more common in hospitalized patients than patients in the community; therefore, this reference group will not be representative of all pandemic (H1N1) 2009 patients, and our results cannot be generalized to community cases. Although sporadic (*14**,**23*) and clustered (*15*) cases of oseltamivir resistance have been reported in communities in several countries, the World Health Organization has not reported widespread community circulation of oseltamivir-resistant pandemic (H1N1) 2009 virus (*24*). In the United Kingdom, more than one third of subtype H1N1 specimens tested for antiviral susceptibility were from patients from the community rather than hospitalized patients. However, only 2 of 45 patients (4.4%) with oseltamivir-resistant virus were from the community, and both cases were treatment induced. The recommendations for antiviral susceptibility testing introduced a second selection bias in this study. Our reference group was found to be representative of patients hospitalized with pandemic (H1N1) 2009 in terms of age and sex. However, the proportion of patients with underlying disease and immunosuppression was lower in the tested controls than in the nontested group. This finding may have led to a slight overestimation of the size of the association between these risk factors and the development of oseltamivir-resistant virus.

In addition, patients who had any complication and those admitted to ICU were overrepresented in our reference group, meaning that the course of the disease was studied among the patients with the most severe concurrent conditions who were hospitalized with pandemic (H1N1) 2009. In addition, because the number of diagnosed cases of oseltamivir-resistant pandemic (H1N1) 2009 virus infection remains limited, any associations should be interpreted carefully. Last, although information on the case-patients with oseltamivir-resistant strains was actively collected, information for the controls with oseltamivir-sensitive strains was voluntarily reported by hospital microbiologists and was therefore subject to potential reporting bias.

In contrast to findings for seasonal influenza, the initial epidemiologic findings of pandemic (H1N1) 2009 in the United Kingdom were that persons <24 years of age were more likely to become infected than persons >65 years of age (*25*). However, in our study, >60% of the infections with oseltamivir-resistant viruses occurred in persons >45 years of age. The high proportion of immunocompromised persons among the resistant cases presumably explains this age difference. In a study done in the United Kingdom, the prevalence of immunocompromised patients increased with age, from 1.5% in children and young adults >7% in persons >70 years of age (*26*).

In this report, 93.5% of the resistant case-patients and 58.9% of the susceptible controls had >1 underlying medical condition. In several other countries, the presence of >1 risk factor was associated with an increased risk for hospitalization (*25**,**27*). As in patients with seasonal influenza, chronic respiratory disease was the most commonly reported underlying medical condition for control patients infected with a susceptible virus. However, 70% of the resistant case-patients were immunosuppressed, and immunosuppression was the only independent variable associated with the presence of an oseltamivir-resistant virus, with most of the case-patients having received oseltamivir therapy before being diagnosed with a resistant strain. These results are consistent with several other reports in which resistance seemed to develop more frequently among severely immunosuppressed patients treated with antiviral drugs (*24**,**28**–**30*). Prolonged virus shedding in the setting of antiviral therapy is known to lead to increased risk for the emergence of oseltamivir-resistant seasonal influenza viruses (*6*). Instances of immunosuppressed patients with prolonged virus shedding have been documented for oseltamivir-resistant seasonal and pandemic (H1N1) 2009 influenza viruses (*31*). In addition, prophylaxis and treatment were recommended for immunocompromised patients during the 2009–10 influenza pandemic.

The clinical features of case-patients infected with oseltamivir-resistant pandemic (H1N1) 2009 virus were similar to those previously described for patients hospitalized during the pandemic (*25**,**27*): fever and cough were the most common symptoms, and ≈30% of the case-patients had gastrointestinal symptoms. Of note, dyspnea was present in 55% of case-patients, which may suggest an early lower respiratory tract infection in these patients. In this study, pneumonia was the main complication reported for patients with oseltamivir-resistant strains and those with oseltamivir-sensitive strains. Pneumonia is a usual complication of seasonal influenza, particularly among immunocompromised patients (*32*). A significant proportion of patients hospitalized with pandemic (H1N1) 2009 were also reported with pneumonia (*27**,**29**,**33*). The ability of the pandemic (H1N1) 2009 virus to replicate in the lungs, as shown in animal models (*34*), may explain the high frequency of this complication in the 2009–10 pandemic. Although the risk for such complications developing was significantly higher among patients with oseltamivir-resistant strains, this result should be interpreted carefully as no information regarding either a possible bacterial co-infection or the time of sampling during the course of illness was available.

Half of the patients infected with an oseltamivir-resistant virus were admitted to the ICU, and approximately one third died. Although the risk for developing more severe outcomes appeared higher among patients with oseltamivir-resistant strains, the multivariate analysis indicated that the presence of an underlying medical condition, especially immunosuppression or chronic respiratory disease, played a more important role in the development of such severe outcomes. In other studies (*27*,*35**–**37*), underlying concurrent conditions correlated with a high risk for ICU admission and death. Immunosuppression has already been described as an important risk factor for ICU admission and death during seasonal influenza outbreaks (*38*). A more severe outcome of pandemic (H1N1) 2009 virus infection among immunocompromised persons was also reported in several studies (*27**,**29**,**30**,**37**,**39*).

In conclusion, clinicians should be aware of the emergence of oseltamivir-resistant pandemic (H1N1) 2009 virus, particularly in immunosuppressed patients. Testing for antiviral resistance is needed, especially among this group, to ensure appropriate antiviral prescribing, minimize the risk for treatment failure, and minimize the risk of person-to-person transmission of a resistant strain. Although the selective pressure of treatment seems to be the most likely mechanism to explain the development of resistant strains, person-to-person transmission has also been demonstrated. To limit the potential for secondary transmission of resistant virus, it is recommended that clinicians check for virus clearance at the end of treatment. Changes in the recommendations of antiviral drug use for immunocompromised patients are already implemented in the United Kingdom. Either zanamivir as monotherapy or oseltamivir combined with zanamivir should be offered as primary treatment for all immunocompromised patients. Although the immune response to vaccine can be lower in some persons, particularly those who are immunosuppressed, influenza vaccination remains the major intervention to protect immunosuppressed patients who are at risk for the development of more severe disease.
